# Undiagnosed Diabetes in Patients Admitted to a Clinical Decision Unit from the Emergency Department: A Retrospective Review

**DOI:** 10.7759/cureus.3390

**Published:** 2018-10-01

**Authors:** Jessica Sop, Mark Gustafson, Clyde Rorrer, Alfred Tager, Frank H Annie

**Affiliations:** 1 Emergency Medicine, Charleston Area Medical Center, Charleston, USA; 2 Emergency Medicine, Charleston Area Medical Center, Charleston , USA; 3 Cardiology, Charleston Area Medical Center, Charleston, USA

**Keywords:** diabetes, clinical decision unit, observation unit, preventative healthcare, screening

## Abstract

Objectives

Diabetes is a debilitating disease that affects the way the body uses or produces insulin. Research evaluating the usefulness in screening patients admitted to a clinical decision unit (CDU) from the emergency department (ED) has been limited.

Methods

A retrospective chart review of patients admitted to a CDU from the ED was performed. Patients included were > 18-year-old who were observed in the CDU, had blood glucose drawn greater than eight hours after admission, and who had not been previously diagnosed with diabetes. Age, sex, and fasting glucose level were collected. The analysis was done to evaluate the percentage of patients undiagnosed and at risk for diabetes mellitus by assessing fasting blood glucose the morning after admission.

Results

Study revealed that 27.8% of the patients analyzed in this study had fasting blood glucose levels meeting or exceeding the diagnostic threshold of 126 mg/dL and could potentially have undiagnosed diabetes.

Conclusion

Screening patients admitted to a CDU from the emergency department identified that 27.8% had fasting plasma glucose levels ≥ 126 mg/dL. Consideration should be made to obtain a fasting blood glucose level in those without a previous diagnosis of diabetes who are observed overnight in a CDU.

## Introduction

Diabetes and its associated complications pose a major health dilemma worldwide. In the United States, the prevalence of diabetes has increased to an estimated 30.3 million cases reported in 2015, affecting 9.4% of the U.S. population [1]. While awareness of the disease is increasing, as many as 7.2 million of these patients remain undiagnosed [1]. The percentage of patients with controlled diabetes has only increased by 1.5% from 1999 to 2012. There is also a significant national disparity in the success of treatment, with some counties in the U.S. having a lack of control as high as 12.3% [2]. The lack of control, high prevalence, and large number of undiagnosed patients place a significant burden on the healthcare system. In 2015, diabetes was the 15th highest contributor to years of life lost and had a global economic burden of $1.3 trillion dollars. This is projected to increase to over $2.1 trillion dollars by 2030 [3]. The cause of this economic burden is not limited to the primary disease process. Diabetes has been found to be an independent risk factor for the development of atherosclerotic heart disease [4]. The American Heart Association (AHA) recognizes diabetes as one of the seven major controllable risk factors for cardiovascular diseases [5]. Diabetes has also been linked to many other complications including stroke, renal disease, skin infections, eye complications, and gastrointestinal disorders [6]. The risks of complications from diabetes may be lowered when blood glucose levels are kept under control.

Early detection of patients with undiagnosed diabetes could potentially decrease complications of the disease process by allowing for earlier treatment. This ultimately could decrease the overall burden the disease has on healthcare. The diagnosis of diabetes can be delayed as patients may be asymptomatic and in the preclinical stage for up to 10 to 12 years, during which testing still has been found to be reliable and can be used to detect diabetes even though patients may be without symptoms [7]. There has been previous literature evaluating the effectiveness of early detection. Feldman et al. evaluated the effectiveness of early detection of diabetes in asymptomatic patients through screening patients in a population in Sweden. They found that the average age of diagnosis of diabetes was 4.6 years earlier for patients who were screen-detected compared to those who were clinically detected. With earlier detection the screen-detected patients had significantly lower all-cause mortality, cardiovascular disease, renal disease, and retinopathy [8]. Herman et al. used a simulation model with data from the ADDITION-Europe study and found that there is an absolute and relative risk reduction with early detection and treatment of diabetes compared to a three or six-year delay in diagnosis [9]. With such a large percentage of patients going undiagnosed and the clear benefits of early detection, further research is needed about the best way to capture this subset of patients.

In the U.S., 62 million Americans have inadequate access to a primary care provider and therefore do not benefit from preventive medicine and screening [10]. In 2014, there were 141.4 million emergency department visits in the United States [11]. The emergency department (ED) may be the only exposure to a clinician some patients may have which represents an opportunity to screen for comorbidities in these patients, such as diabetes. Previous literature has found that screening for diabetes in the ED setting by using random blood glucose or HbA1c levels can potentially identify patients with undiagnosed diabetes or prediabetes that could benefit from outpatient follow-up [12-18]. These studies are limited as a large percentage of patients presenting to the ED are not fasting therefore random blood glucose levels in this setting could potentially miss patients whose random glucose level is below 200 mg/dL.

Patients admitted to a clinical decision unit (CDU) from the emergency department for observation could also represent a cohort of patients where screening for diabetes may be beneficial. Wiederhold et al. looked at patients admitted to a low risk chest pain unit from the ED and found that 23.7% of patients with no known history of diabetes had HbA1C levels greater than 5.9% and 30.5% had an elevated random blood glucose level [19]. Patients admitted to a CDU often are observed overnight for further testing and are routinely placed NPO (nothing by mouth) [20]. This represents an opportunity to evaluate the morning laboratories for a fasting blood glucose >126 mg/dL. These patients could then potentially undergo diabetes education, initiate treatment, and have appropriate follow-up arranged prior to discharge.

The purpose of this study was to determine whether or not the patient population admitted to a CDU from the ED is at risk for undiagnosed diabetes by analyzing fasting blood glucose levels the morning following admission.

## Materials and methods

This was a retrospective chart review of patients’ records observed over a one-year period in patients admitted to the CDU from July 1, 2013 to June 30, 2014 following ED evaluation. This study took place at Charleston Area Medical Center (CAMC) in Charleston, West Virginia. All patients who were over 18 years of age without a previous diagnosis of diabetes mellitus were admitted into the study. Patients who did not have labs drawn or have labs drawn less than eight hours after admission were excluded from the study. All included patients had labs drawn eight hours following CDU admission and their fasting blood glucose level was collected. The patients’ demographics (age, sex) were also collected. This study was approved by our institutional review board.

## Results

**Figure 1:**
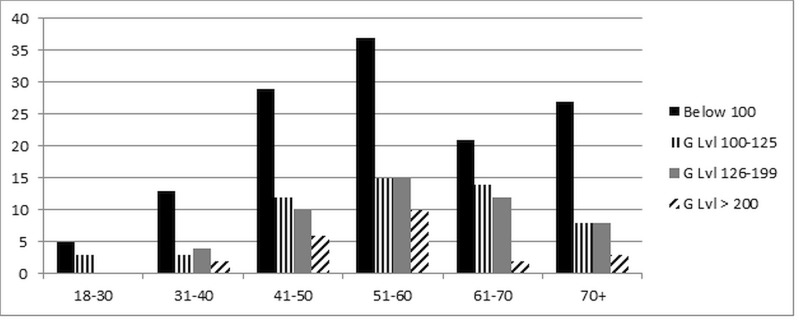
Fasting Blood Glucose Levels (By Age). G Lvl: Glucose Level

There were 1,494 patients who were admitted to the CDU during the data collection period who were aged 18 and over with no previous diagnosis of diabetes mellitus. Of these, 1,047 were admitted through the ED. A total of 259 of these patients (146 females, 113 males) had blood drawn > 8 hours after their presentation time to measure fasting blood glucose and therefore were included in the study. Characteristics for the 259 study patients with no previous diabetes diagnosis are shown in Figure 1 and Table 1.

**Table 1:** Fasting Blood Glucose Levels (By Age).

	Fasting Blood Glucose Level (mg/dL)				
Age (yrs)	< 100	100-125	126-199	> 200	Total
18-30	5	3			8
31-40	13	3	4	2	22
41-50	29	12	10	6	57
51-60	37	15	15	10	77
61-70	21	14	12	2	49
70+	27	8	8	3	46
Total	132	55	49	23	259

Fifty-five patients (31 females, 24 males) were found to have glucose levels from 100 to 125 mg/dL. Seventy-two patients (45 female, 27 male) were found to have glucose levels ≥ 126 mg/dL. Twenty-three of those 72 patients (14 male, nine female) were found to have glucose levels ≥ 200 md/dL. These results are demonstrated in Table 2. 27.8% of patients admitted to the CDU through the ED who had fasting labs drawn had a fasting blood glucose level >126 mg/dL and were at risk for diabetes. An additional 21.2% of patients were at risk for pre-diabetes.

**Table 2:** Fasting Blood Glucose Levels (By Gender).

	100-125	126-199	> 200	Total (includes < 100)
Male	24 (43.64%)	13 (26.53%)	14 (60.87%)	113 (43.63%)
Female	31 (56.36%)	36 (73.47%)	9 (39.13%)	146 (56.37%)
Total	55 (21.2%)	49 (19%)	23 (8.8%)	259 (100%)

## Discussion

Diagnosis of diabetes in the ED can be difficult due to the diagnostic guidelines set forth by the American Diabetes Association (ADA). According to the guidelines the diagnosis of diabetes is made by satisfying one of the following criteria: (1) HbA1C ≥ 6.5%, (2) a fasting blood glucose (FPG) ≥ 126 mg/dL, (3) two-hour blood glucose ≥ 200 mg/dL during an oral glucose tolerance test, or (4) a random blood glucose of 200 mg/dL or higher in a patient with classic symptoms of hyperglycemia or hyperglycemic crisis [21]. The ADA recommends repeat testing for confirmation in the absence of unequivocal hyperglycemia. Also, the diagnosis of diabetes can be made if two different tests are both above the diagnostic threshold. Screening for diabetes in the ED by evaluating blood glucose levels is limited to the fact that most patients are not fasting. Therefore, obtaining a fasting glucose cannot be routinely used in this setting for screening purposes. Also, HbA1c is rarely ordered in the ED setting as it does not change management acutely. The most commonly encountered diagnostic criteria is number four, as patients that are symptomatic with a random blood glucose >200 mg/dL can be detected. These patients could be referred for outpatient follow-up for confirmatory testing but there is not an option to diagnose them with a fasting glucose in the ED to formally diagnose diabetes and start management on the day of their visit. As noted previously there have been several studies identifying that there are a large percentage of patients that visit the ED that could be at risk for diabetes but without ordering a HbA1c or follow-up testing these patients may get lost to follow up.

Patients admitted to a CDU from the ED are often admitted overnight and have further testing performed. Patients can be placed NPO after midnight and have fasting labs ordered in the morning along with a HbA1c. This setting offers an opportunity to capture more undiagnosed patients with diabetes. Patients meeting two criteria in the CDU could be diagnosed with diabetes, receive education, start treatment, and have close follow-up arranged. Our study evaluated what percentage of patients admitted to our CDU from the ED were at risk for diabetes by the above criteria and found that 27.8% of the patients in this study had glucose levels meeting one of the criteria for the diagnosis of diabetes. Additionally, 21.2% of patients were found to have glucose levels in the prediabetic range.

The previous study by Wiederhold et al. found a similar percentage of 23.7% of patients admitted to their chest pain unit who had HbA1C >5.9% [19]. This represents almost a quarter of patients who are admitted to the CDU who may not otherwise receive preventative care testing that could potentially be diagnosed with diabetes in the CDU by using a HbA1C coupled with a fasting plasma glucose level. The benefit of combining a fasting plasma glucose level with a HbA1C on all patients admitted to the CDU could be substantial, as this would not only screen for diabetes but also could potentially diagnose diabetes if both criteria are positive. This would allow for immediate initiation of treatment, diabetes counseling, and arrangement for close follow-up. The results of our study are beneficial to providers who treat patients who are admitted to an observation unit or CDU from the ED. The implications of adding a morning fasting plasma glucose level to these patients’ workup are profound and should be considered in this setting.

Limitations

NPO status is routinely ordered for patients observed in our CDU. A limitation to our study is that while NPO order status on all patients can be confirmed, adherence to these orders by the patients is not possible to prove due to the retrospective nature of this study. Also, a requirement of enrollment in our retrospective study was that patients admitted to the CDU had to have labs drawn on the morning following admission. Only 24.7% of patients admitted to the CDU met this requirement.

## Conclusions

Consideration should be made to obtaining both a fasting blood glucose and HbA1C level in patients admitted to a CDU from the ED who do not have a previous diagnosis of diabetes. This would allow for early detection and initiation of therapy in patients with diabetes who are undiagnosed and prevention of associated comorbidities and complications.
